# Role of CD44^high^/CD133^high^ HCT-116 cells in the tumorigenesis of colon cancer

**DOI:** 10.18632/oncotarget.7084

**Published:** 2016-01-30

**Authors:** Jin-Yong Zhou, Min Chen, Long Ma, Xiaoxiao Wang, Yu-Gen Chen, Shen-Lin Liu

**Affiliations:** ^1^ Central Laboratory, The Affiliated Hospital of Nanjing University of Chinese Medicine, Jiangsu Province Hospital of Traditional Chinese Medicine, Nanjing, China; ^2^ Department of Internal Medicine, The Affiliated Hospital of Nanjing University of Chinese Medicine, Jiangsu Province Hospital of Traditional Chinese Medicine, Nanjing, China; ^3^ Department of Urology, The Affiliated Hospital of Nanjing University of Chinese Medicine, Jiangsu Province Hospital of Traditional Chinese Medicine, Nanjing, China; ^4^ Department of Medical Science Research, The Affiliated Hospital of Nanjing University of Chinese Medicine, Jiangsu Province Hospital of Traditional Chinese Medicine, Nanjing, China; ^5^ Department of Colorectal Surgery, The Affiliated Hospital of Nanjing University of Chinese Medicine, Jiangsu Province Hospital of Traditional Chinese Medicine, Nanjing, China; ^6^ Department of Oncology, The Affiliated Hospital of Nanjing University of Chinese Medicine, Jiangsu Province Hospital of Traditional Chinese Medicine, Nanjing, China

**Keywords:** CD133, CD44, cancer stem cells (CSCs), colorectal cancer, biomarkers

## Abstract

This study aimed to explore cell surface biomarkers related to cancer stem cells (CSCs) and their role in the tumorigenesis of colon cancer. Various colon cancer cell lines were screened for CD133 and CD44 expression. CD44^high^/CD133^high^ and CD44^low^/CD133^low^ cells were separately isolated by Fluorescence-Activated Cell Sorting (FACS). The cell proliferation, colony formation, cell cycle characteristics, and tumorigenic properties in CD44^high^/CD133^high^ and CD44^low^/CD133^low^ cells were investigated through *in vitro* experiments and *in vivo* tumor xenograft models. The expression profiles of stem cell-related genes were examined by RT-PCR. With HCT-116 cells, flow cytometry analysis revealed that CD44^high^/CD133^high^ cells had higher proliferation potency than CD44^low^/CD133^low^ cells. Compared to CD44^low^/CD133^low^ cells, CD44^high^/CD133^high^ cells had more stem cell-related genes, and displayed increased tumorigenic ability. In summary, CD44^high^/CD133^high^ cells isolated from HCT-116 cells harbor CSC properties that may be related to the tumor growth of colon cancer. These results suggest that CD44 and CD133 could be strong markers of colorectal cancer stem cells.

## INTRODUCTION

Colorectal cancer is the third most common cause of cancer death in the world [1, 2]. There has been a decline in incidence in the past two decades due to better cancer screening measures [3]. Despite these actions, the morbidity of the disease has not decreased as drastically as those with other types of cancer. This could be changed, however, with the expanding knowledge of the biology of colon cancer cells, particularly with the new subset of cells referred to as cancer stem cells (CSCs). Currently, in addition to surgery, fluorouracil (5-FU) in combination with other anti-cancer agents is used as the standard first line chemotherapy based on NCCN guidelines. Because the heterogeneity of colorectal tumors calls for individualized treatments [4, 5], understanding the characteristics of colon CSCs may lead to revolutionary findings in targeted therapeutic strategies.

For years, cancer cells were thought to be stochastic in nature and originating from mutations in normal adult cells. However, a sub-population of cells, the CSCs, was found to display stem cell characteristics that influence tumorigenesis. These CSCs have various cancer-promoting characteristics such as self-renewal, differentiation, chemoresistance, and metastatic potential [5–10]. The exact mechanism of tumorigenicity with which CSCs form cancer is unknown, but the complexity of the microenvironment that cells thrive in is gradually being unveiled. Past scientists have viewed stemness of cells as a combination of intrinsic cell properties as well as circumstantial conditions [11, 12]. CD133 is one of the first stemness markers used to identify CSCs [6, 7] and its expression is a strong predictor of declining prognosis, as high CD133 levels conversely relate to low 5-year overall survival (OS) and disease free survival (DFS) rate in cancer patients [13–15]. Other putative markers include CD44, CD24, LGR5, ALDH, and CD44v6, though not all are definitive markers that differentiate CSCs from normal adult stem cells, and inconsistent level of these markers are presented across varying stages of the tumor [13, 16].

CD133 and CD44 are well-recognized stem cell biomarkers expressed in colorectal cancer [3, 17]. High CD133 expression is especially correlated with tumorigenicity, metastasis, and worse prognosis [17]. It has a strong potential as a target for drug therapies, since many colon cancer cell lines will express this marker [18, 19]. The role of CD44, a hyaluronic receptor, is to promote cell-adhesion and assembly of cell surface growth factors, specifically in the maintenance of cell-matrix interactions [17]. When up-regulated, CD44 increases tumor growth and anti-apoptotic property [19]. In Sahlberg's experiment, three colon cancer cell lines HT-29, HCT-116, and DLD-1 expressed different amounts of CD133, CD44, and other markers. Side population cells extracted from the SW480 cell lines showed protein expression of CD44 and other markers of chemoresistance [17, 20, 21].

Previous reports showed the presence of these biomarkers in many colon cancer cell lines, yet only a few research groups expand their studies to divulge to their exact functions. In this study, we identify that a colon cancer cell line, HCT-116 yield a high percentage of CD44+/CD133+ cells and look further into their relevance to tumor growth property in colon cancer.

## RESULTS

### Screen for CD44 and CD133 expressed cells in various colorectal cancer cell lines

**Figure 1:**
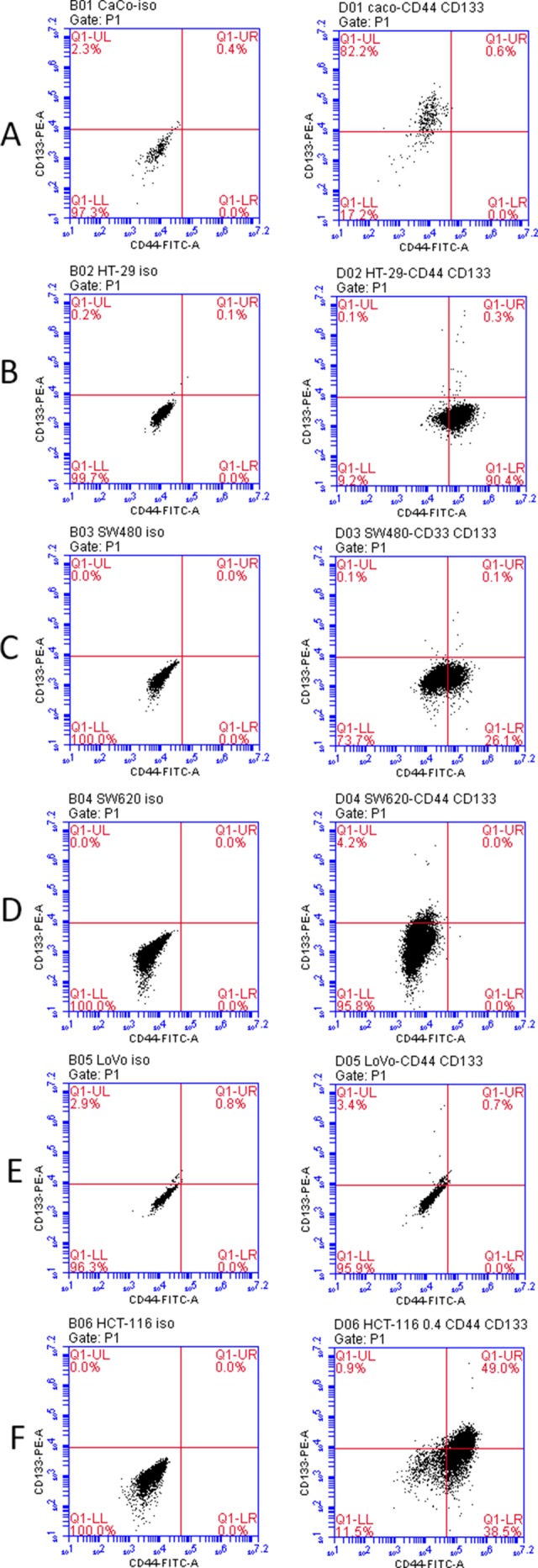
Co-expression of CD44 and CD33 in variable colorectal cancer cell lines as determined by flow cytometry Left column: iso control. Right column: CD44-FITC and CD133-PE staining. (**A**) Caco2 cells (**B**) HT-29 cells (**C**) SW480 cells (**D**) SW620 cells (**E**) LoVo cells (**F**) HCT-116 cells.

All cancer cells express varying levels of CD44 and CD133. To delineate CD44 and CD133 expression in colon cancer cells, we used flow cytometry to measure the level of expression of these surface molecules in various colon cancer cell lines. We examined Caco2 cells, HT-29 cells, SW480 cells, SW620 cells, LoVo cells, and HCT-116 cells. The number of cells that expressed the markers in each cell line is visualized in Figure 1. It was found that HCT-116 cells had the highest percentage of CD44^high^/CD133^high^ cells. HCT-116 cells were used for further study.

### Isolation of CD44^high^/CD133^high^ and CD44^low^/CD133^low^ cells

**Figure 2:**
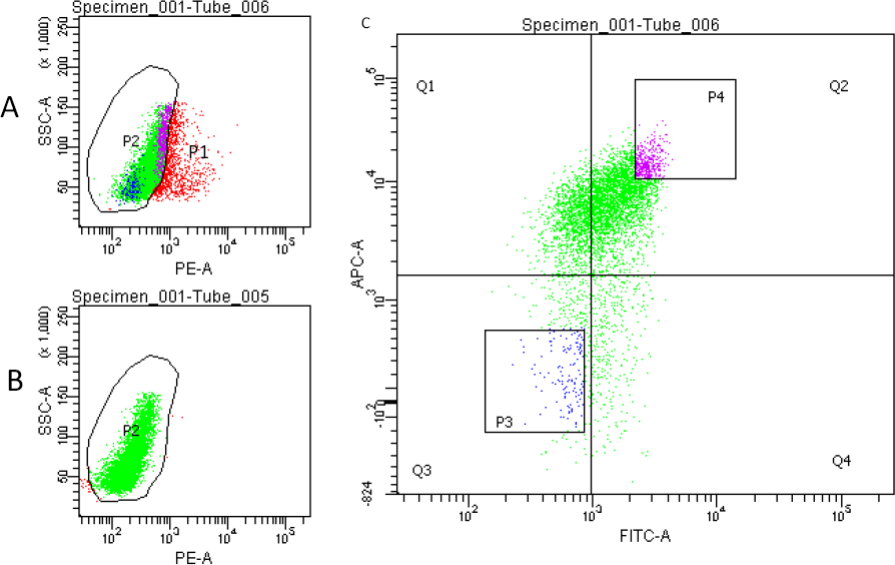
Flow cytometry sorting of HCT-116 cells (**A**) Cells were stained using CD44-FITC, CD133-APC and PI to separate live cells (P2, green) and dead cells (red). (**B**) Control staining using CD44-FITC and CD133-APC. (**C**) Fluorescence-Activated Cell Sorting (FACS). P3: CD44^low^/CD133^low^ cells. P4: CD44^high^/CD133^high^ cells.

As mentioned previously, HCT-116 displayed a high percentage of CD44^high^/CD133^high^ cells. Therefore, we chose to isolate cells that are CD44^high^/CD133^high^ and CD44^low^/CD133^low^. Cells were isolated by Fluorescence-Activated Cell Sorting (FACS), followed by serum-free medium (SFM) culture. PI was used as a nuclear staining. Double staining was achieved using CD44-FITC (green color) and CD133-APC (red color). Figure 2 depicts the flow cytometry data following staining and sorting. Sorting the cells successfully resulted in isolation of both CD44^high^/CD133^high^ and CD44^low^/CD133^low^ cells (approximately 3–5% of the total cells each).

### Cell cycle analysis

**Figure 3:**
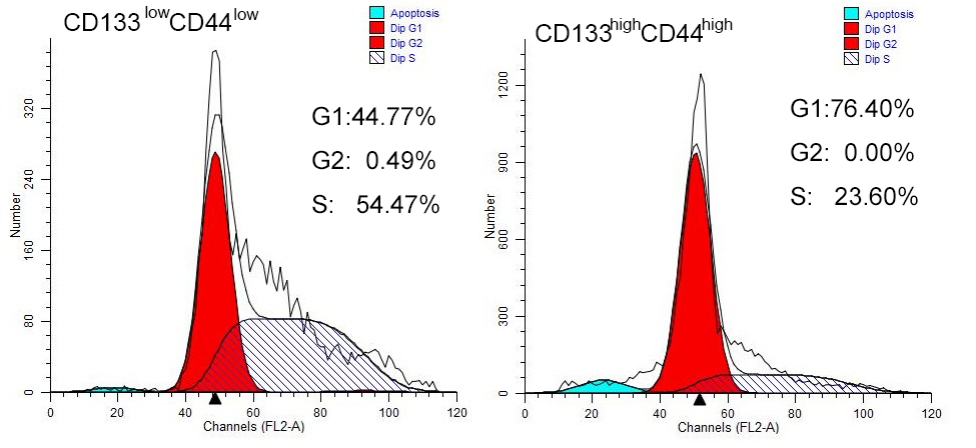
Cell cycle analysis of CD44^high^/CD133^high^ and CD44^low^/CD133^low^ cells in HCT-116 cell line More cells are in G0/G1 phase in the CD44^high^/CD133^high^ cell population. (**A**) CD44^low^/CD133^low^ cells (**B**) CD44^high^/CD133^high^ cells have higher growth capability.

To evaluate the growth capability of cells, both populations of cells were further examined by using cell cycle analysis to evaluate the number of cells represented in each specific replication phase. Figure 3 shows that less CD44^low^/CD133^low^ cells were found to be in the G0/G1 phase, indicating lower growth capability than the CD44^high^/CD133^high^ cells. Both the higher expression of CD44 and CD133 may indicate higher exponential replication, and therefore faster tumor growth.

### Differential expression of stem cell markers in CD44^high^/CD133^high^ and CD44^low^/CD133^low^ cells

**Figure 4:**
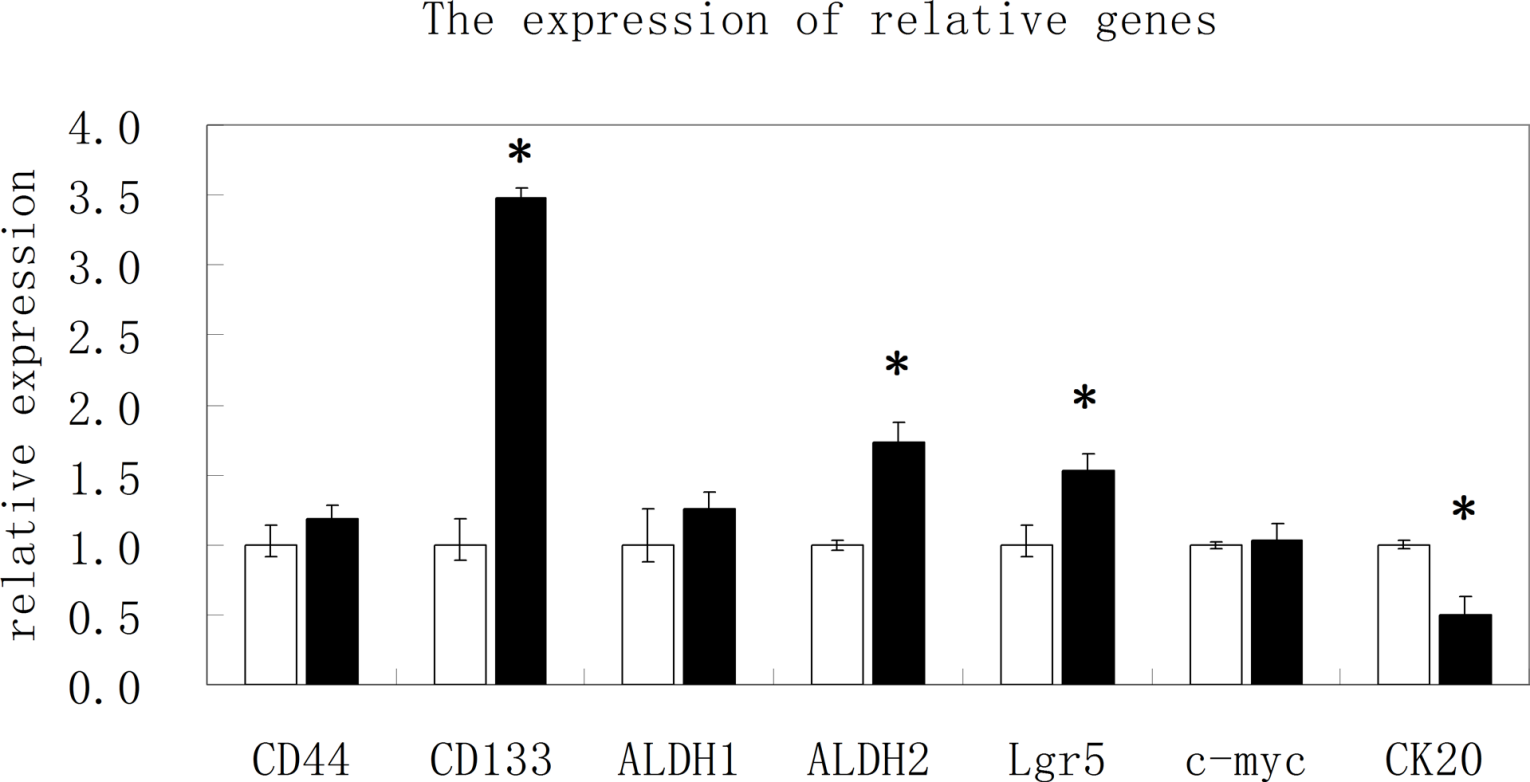
The results of real time PCR for various stem cell markers The relative expression of mRNA for CD44, CD133, ALDH1, ALDH2, Lgr5, c-myc and CK20 in CD44^high^/CD133^high^ and CD44^low^/CD133^low^ cells.

To understand the difference of expression of cancer stem cell markers in CD44^high^/CD133^high^ and CD44^low^/CD133^low^ cells, expressions of various other stem cell markers in addition to CD44 and CD133 were measured in these two cell populations. RNAs were isolated from the two populations of cells. Fluorescent quantitative real-time PCR was performed to investigate the mRNAs of these cells. The results in Figure 4 suggest that genes for stem cell markers CD44, CD133, LGR5, and ALDH1 and ALDH2 are overexpressed in CD44^high^/CD133^high^ cells, while a gene for differentiated cells CK20 was overexpressed in CD44^low^/CD133^low^ cells.

### Cell proliferation and colony formation properties

**Figure 5:**
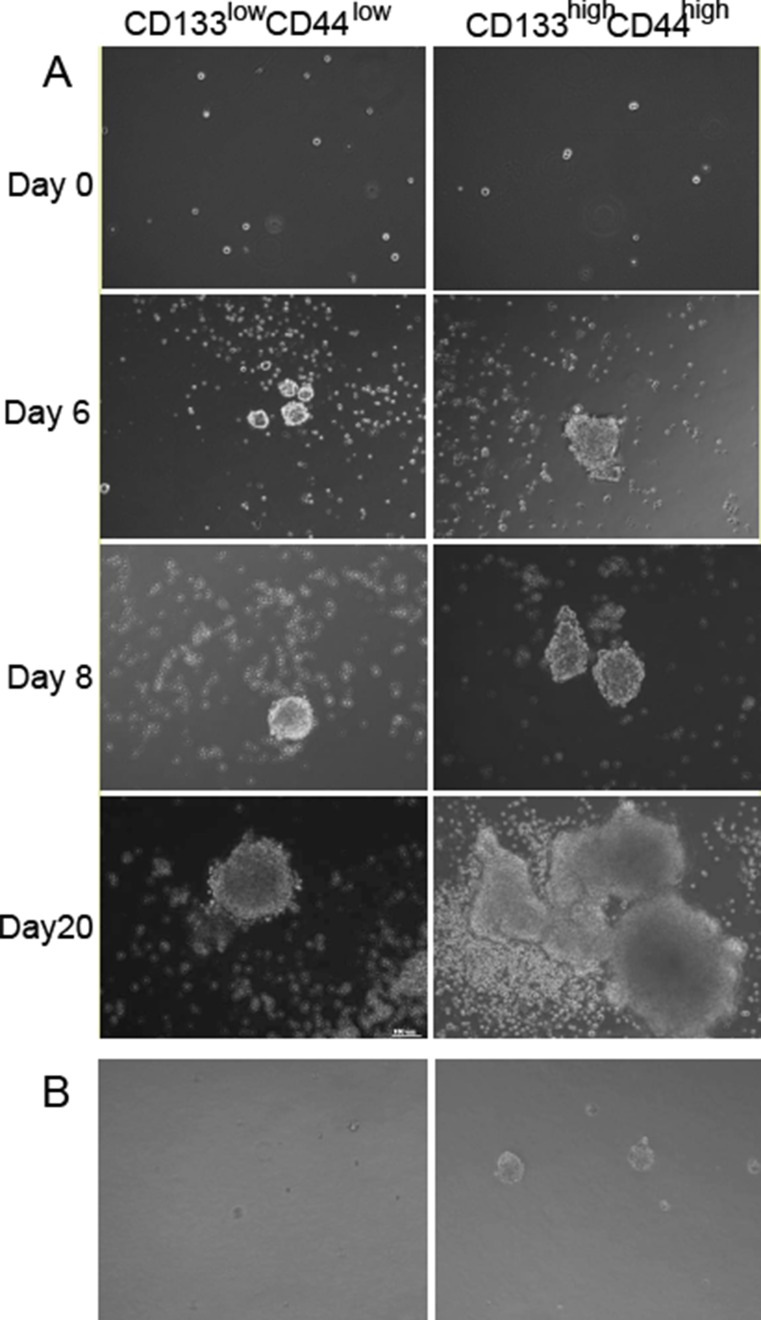
Formation of stem cell sphere after seeding the sorted CD44^high^/CD133^high^ and CD44^low^/CD133^low^ cells Left: CD44^low^/CD133^low^ cells. Right: CD44^high^/CD133^high^ cells (**A**) Cells cultured in low adhesion 6-well plates. (**B**) Cells seeded on low melting point agarose coated plates.

One of the characteristics of stem cells is the formation of spheroidal cell mass. To investigate the growing characteristics of CD44^high^/CD133^high^ and CD44^low^/CD133^low^ cells, both populations of cells were sorted and cultured in serum-free medium (SFM) in low adhesion 6-well plates. The cells were observed with images captured at different time points (0, 6, 8, 20 days) as shown in Figure 5A. It was found that by Day 6 there was a noticeable difference in colony formation ability and size between the CD44^high^/CD133^high^ and CD44^low^/CD133^low^ cells. Irregular formation of the colonies by Day 20 suggests an increase in cell differentiation in the CD44^high^/CD133^high^ cells. When CD44^high^/CD133^high^ and CD44^low^/CD133^low^ cell populations were seeded on plates coated with low melting point agarose and cultured for 8 days, the formation of colony was much better in the CD44^high^/CD133^high^ group than in CD44^low^/CD133^low^ group (Figure 5B).

### Tumor forming property *in vivo*

**Figure 6:**
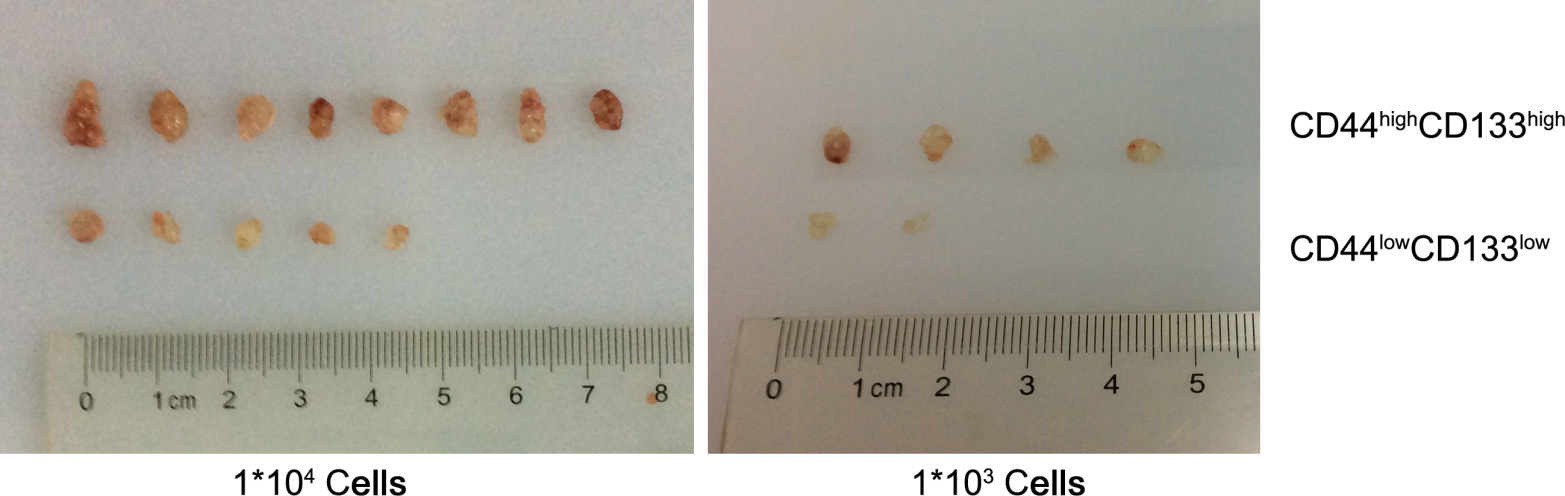
The formation of tumors between CD44^high^/CD133^high^ and CD44^low^/CD133^low^ HCT-116 cells in mice models

**Figure 7:**
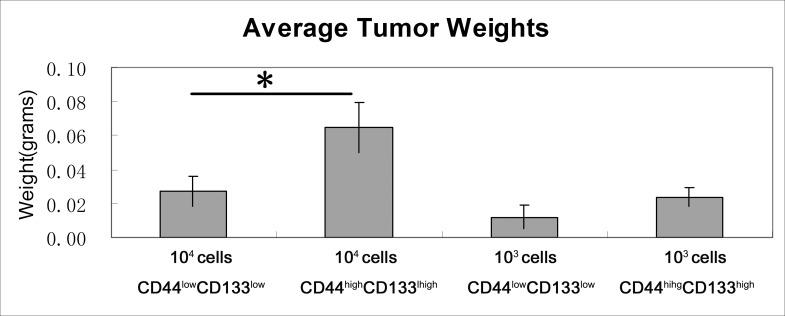
Tumor weights in CD44^high^/CD133^high^ and CD44^low^/CD133^low^ populations Mice that did not form a tumor were not included in the statistics. *P < 0.05.

To investigate the tumorigenic properties of CD44^high^/CD133^high^ and CD44^low^/CD133^low^ cells, we induced tumor models in mice. Cells were sorted by FACS and injected into the right flank of 6-week-old Balb/c nude mice at two concentrations: 1 × 10^4^ and 1 × 10^3^. After 19 days, animals were sacrificed and the tumors were dissected and weighted. Figure 6 visualizes the various tumor sizes as a result. The concentration of cells injected correlates positively to the percent of tumor formation. When mice were injected with 10^4^ or 10^3^ cells there was a distinction between the CD44^high^/CD133^high^ and CD44^low^/CD133^low^ cells in their tumor forming potential. The variation in tumor bulk can be observed as well. Figure 7 shows that in each cell concentration group, the average tumor weight was higher in the CD44^high^/CD133^high^ groups, and this increase was significant in the 10^4^ cell injection group.

## DISCUSSION

The formation of cancer was once thought to be an accumulation of random mutations in normal adult cells. This is quickly being replaced by a new theory on the organized manner of tumor cell proliferation, particularly with the cancer stem cells [3, 5, 10, 16]. CSCs are a subset of cells that can self-regenerate, differentiate, and facilitate tumor proliferation [5–10, 25]. The objectives of the study were to identify the common cell surface biomarkers in colon cancer stem cells and the activities relevant to these markers. In this study, CD133 and CD44 expressed cells were found to be highest in the HCT-116 cells, a metastatic colon cancer cell line [26]. Past studies have found a correlation between the percentage of CD133 positive cells in CSCs and tumor aggressiveness [27–29], cell invasion [30], and poor prognosis [31, 32]. The level of CD44 expression is associated with more cancer metastasis and lower overall patient survival [33]. Therefore, the significant levels of CD133 and CD44 found in HCT-116 cells are concurrent with the aggressive cancer properties such as tumor proliferation and metastasis that these cells display in colon cancer. In a recently published paper [17], high presence of CD44^high^/CD133^high^ cells were observed in both HT-29 and HCT116 cell lines. We did not find high presence of CD44^high^/CD133^high^ cells in HT-29 cell line. One possible explanation might be that instead of using single antibody for CD44 and CD133, researchers in this group used two different specific antibodies for each biomarker, so they were able to pick up more CD44^high^/CD133^high^ cells. Nevertheless, CD133 and CD44 could be strong biomarkers for the identification of CSCs in colon cancer.

The cell cycle analysis revealed that cells that expressed CD133 and CD44 were primarily in the G0/G1 phase, indicating that they have higher growing capability than the cells that were CD44^low^/CD133^low^. In a study by Kawamoto et al., CD133 cells were found to have faster tumor formation *in vitro* and *in vivo* [34].

Other stem cell markers were observed in the CD44^high^/CD133^high^ cells. CD44^high^/CD133^high^ cells also showed more overexpression of LGR5 and ALDH2. Previous research showed similar expression levels of these biomarkers in CSCs [18, 35]. These markers are also related to the downstream molecular pathways that carry out tumorigenic activities such as Wnt, Notch, and BMP-1 [18, 36]. In addition, CD47 (the integrin-associated protein) is a “don't eat me” molecule of the cell surface immunoglobulin superfamily that is implicated to enable tumors to escape innate immune system surveillance through the evasion of phagocytosis. There were reports that circulating tumor cells (CTCs) overexpressed CD47. Since HCT116 cells are highly metastatic, it might overexpress CD47 to escape the endogenous “eat me” signals. Future study will be perform to investigate the expression of CD47 in CD44^high^/CD133^high^ and CD44^low^/CD133^low^ cells. Differentiation of CSCs is still an area of vast unknown. There are conflicting findings on this matter. Since the initial discovery of CD133 cells, it was found that CSCs could eventually differentiate into a heterogeneous population of tumor cells [3, 6, 7]. However, some researchers have claimed that HCT-116 is a cell line that consists mostly of cells that no longer have the ability to differentiate [25], and that it is a cell line that is vital to tumor aggressiveness and cell invasion [25, 26, 30]. This study's results correspond to those of Xiong et al. which demonstrated that the Side Population (SP) cells of colon cancer can spontaneously differentiate into SP and non-SP cells [20]. The CD44^high^/CD133^high^ cells that were cultured in serum free medium for 20 days had shifted shapes from spheroid to polygonal. Though the observation is preliminary, it may suggest that CD44^high^/CD133^high^ cells maintain the ability to differentiate and grow heterogeneous tumors.

This finding is further supported by the *in vivo* tumor formation study in mice. CD44^high^/CD133^high^ cells had more frequent tumor formation rate in mice. The tumor weight was also higher in CD44^high^/CD133^high^ cells in every concentration group, significantly so in the 1 × 10^4^ cells group. These results suggest CD44^high^/CD133^high^ cells have higher tumor forming potential than CD44^low^/CD133^low^ cells.

There are several limitations in this study that could be addressed in future research. Due to small sample size, the results of the study can only support previously reported conclusions in similar experiments. Expanding on the *in vivo* and *in vitro* testing of the tumor formation can not only further confirm the distinct tumorigenesis rates between CD44^high^/CD133^high^ and CD44^low^/CD133^low^ cells, but may also allow the observation of the timeline of tumor development. Additional studies may be conducted to appreciate the overlapping functions of various CSC biomarkers. We planned to use RNAi technology to down regulate CD133 and CD44 in colon cancer cells to see if it decease the tumorigenic capacity. In addition, we will collect colon cancer patient samples and investigate the expression level of CD133 and CD44. Explore the correlation of expression of CD133/CD44 and the clinicopathological characteristics.

As the knowledge of colon cancer biology grows, it also exposes a myriad of new targets for novel oncotherapy. In this study, CD44 and CD133 were shown to have significant potential as two biomarkers for colon cancer stem cells. A better understanding of colon cancer stem cell specific biomarkers allow for selective drug toxicity against tumor cells, earlier detection and treatment of colon cancer, and a proactive approach to preventative care for colon cancer.

## MATERIALS AND METHODS

### Fluorescence-activated cell sorting (FACS) of cells

Colon cancer cell lines Caco, HT-29, SW480, SW620, LoVo, and HCT-116 were purchased from Shanghai Institute of Biochemistry and Cell Biology (Chinese Academy of Sciences, China). To analyze the expression of CD44 and CD133 in different lines, cells were routinely cultured, harvested, trypsin-digested and re-suspended in stain buffer (1 × 10^6^ cells in 80 μl). Cells were then treated with 20 μl FcR Blocking Reagent for 15 min, and incubated with antibodies (human anti-CD44-FITC and human anti-CD133-PE, Miltenyi Biotec, San Diego, CA, USA) for 30 min. After staining, cells were subjected to flow cytometry for analysis using BD Accuri C6 (CA, USA).

To isolate CD44^high^/CD133^high^ and CD44^low^/CD133^low^ populations in the HCT-116 line, cells were trypsinized and blocked with FcR Blocking Reagent. Propidium iodide staining was applied to exclude the dead cells. Live cells were incubated with antibodies (human anti-CD44-FITC and human anti-CD133-APC, Miltenyi Biotec, Auburn, USA) for 30 min. CD44^low^/CD133^low^ and CD44^high^/CD133^high^ cells were sorted by a cell sorter (BD FACSARIA III, CA, USA).

For cell cycle analysis, CD44^high^/CD133^high^ and CD44^low^/CD133^low^ cells were stained with propidium iodide and analyzed using a flow cytometer (BD Calibur, CA, USA) and ModFit software (Verity, Maine, USA).

### Assessment of the expression of cancer stem cell markers by RT-PCR

Measurements of different cancer stem cell markers using quantitative RT-PCR are described previously [22–24]. Total RNAs were extracted from isolated CD44^high^/CD133^high^ and CD44^low^/CD133^low^ cells using PureLink Kit (Life technologies, MA, USA). RNAs were reversely transcribed to cDNAs (Primescript RT reagent Kit with gDNA Eraser, Takara, China). The primers for human CD44, CD133, ALDH1, ALDH2, Lgr5, c-myc, CK20 and beta-actin (ACTB) were designed using Primer Express and are listed in Table 1. Expressions of target genes were analyzed by ABI 7500 fast real time PCR System and Power SYBR Green PCR Master Mix (Life Technology, MA, USA). The optimized concentrations for real-time PCR were 0.2 μM for both primers in a 10 μl reaction volume. Human ACTB expression was used as an internal control. Each sample was tested in triplicate. The reaction was as follows: 50°C for 20 sec, 95°C for 10 min, followed by 40 cycles of 95°C for 15 sec and 60°C for 1 min, then 95°C for 15 sec. Cycle threshold (Ct) values were obtained graphically for the target genes and ACTB. The difference in Ct values between ACTB and target genes were represented as ΔCt values. ΔΔCt values were obtained by subtracting ΔCt values of control samples from those of treated samples. The relative fold change in gene expression was calculated as 2^−ΔΔCt^.

**Table 1:** Primers of cancer stem cell markers

Gene	Primers sequence 5(5′–3′)	Length of PCR product (BP)
ACTB-F	GGCCAACCGCGAGAAGAT	134
ACTB-R	CGTCACCGGAGTCCATCA	
CD44-F	TGAATATAACCTGCCGCTTTG	136
CD44-R	GCTTTCTCCATCTGGGCCAT	
CD133-F	GAGTCGGAAACTGGCAGATAGCA	113
CD133-R	ACGCCTTGTCCTTGGTAGTGTTG	
ALDH1-F	AGCCTTCACAGGATCAACAGA	124
ALDH1-R	GTCGGCATCAGCTAACACAA	
ALDH2-F	ACCTGGTGGATTTGGACATGGTCC	168
ALDH2-R	TCAGGAGCGGGAAATTCCACGGA	
Lgr5-F	GGTCGCTCTCATCTTGCTCA	171
Lgr5-R	GCCACAGGGCAGTTTAGGAT	
c-myc-F	CACCAGCAGCGACTCTGA	102
c-myc-R	GATCCAGACTCTGACCTTTTGC	
CK20-F	ATGGATTTCAGTCGCAGAAGC	170
CK20-R	CTCCCATAGTTCACCGTGTGT	

### Colony formation assay

Assorted CD44^high^/CD133^high^ and CD44^low^/CD133^low^ cells were seeded in six-well plates (1 × 10^4^ cells per well) and grown in DMEM/F-12 serum-free medium (Gibco, MA, USA) that contains 20 ng/ml EGF, 10 ng/ml bFGF (Peprotech, NJ, USA), 1% B27, 10 ng/ml LIF (life technology, MA, USA), 5 μg/ml insulin, 1 ng/ml hydrocortisone, and 4 μg/ml heparin sodium (Sigma, St. Louis, MO, USA). Medium was replenished every 6 days. To evaluate the ability of colony formation for different cells, images were taken at 0, 6, 8, and 20 days after culture using an inverted microscope (CKX41, Olympus, Japan).

Assorted CD44^high^/CD133^high^ and CD44^low^/CD133^low^ cells were also plated in low melting agarose plates (upper layer 0.6% agarose, bottom layer: 3% agarose). Images were taken under the inverted Olympus microscope at 8 days after plating.

### *In vivo* tumor formation assay

All animal experiments in this study were approved by the IACUC of the affiliated hospital of Nanjing University of Chinese Medicine. Assorted CD44^high^/CD133^high^ and CD44^low^/CD133^low^ cells were counted, suspended in PBS, and inoculated subcutaneously (1 × 10^3^ and 1 × 10^4^, cells per mice, 3 × 7 mice per group) into the right flank of 6-week-old Balb/c nude mice (purchased from SLAC Laboratory animal, Shanghai, China). Animals were sacrificed at 19 days after surgery. Tumor sizes and weights were measured for analysis.
